# Predicting Residue-Residue Contacts and Helix-Helix Interactions in Transmembrane Proteins Using an Integrative Feature-Based Random Forest Approach

**DOI:** 10.1371/journal.pone.0026767

**Published:** 2011-10-28

**Authors:** Xiao-Feng Wang, Zhen Chen, Chuan Wang, Ren-Xiang Yan, Ziding Zhang, Jiangning Song

**Affiliations:** 1 State Key Laboratory of Agrobiotechnology, College of Biological Sciences, China Agricultural University, Beijing, China; 2 State Engineering Laboratory for Industrial Enzymes and Key Laboratory of Systems Microbial Biotechnology, Tianjin Institute of Industrial Biotechnology, Chinese Academy of Sciences, Tianjin, China; 3 Department of Biochemistry and Molecular Biology, Faculty of Medicine, Monash University, Melbourne, Victoria, Australia; Purdue University, United States of America

## Abstract

Integral membrane proteins constitute 25–30% of genomes and play crucial roles in many biological processes. However, less than 1% of membrane protein structures are in the Protein Data Bank. In this context, it is important to develop reliable computational methods for predicting the structures of membrane proteins. Here, we present the first application of random forest (RF) for residue-residue contact prediction in transmembrane proteins, which we term as TMhhcp. Rigorous cross-validation tests indicate that the built RF models provide a more favorable prediction performance compared with two state-of-the-art methods, i.e., TMHcon and MEMPACK. Using a strict leave-one-protein-out jackknifing procedure, they were capable of reaching the top *L*/5 prediction accuracies of 49.5% and 48.8% for two different residue contact definitions, respectively. The predicted residue contacts were further employed to predict interacting helical pairs and achieved the Matthew's correlation coefficients of 0.430 and 0.424, according to two different residue contact definitions, respectively. To facilitate the academic community, the TMhhcp server has been made freely accessible at http://protein.cau.edu.cn/tmhhcp.

## Introduction

Proteins are the building blocks of life. One fourth to one third of them are membrane proteins located in the bilayer lipids [1], [2]. Membrane proteins play important roles in various life processes and possess many complex physiological functions, such as signal transduction, energy generation, metabolic transport and cell recognition. They are also important drug targets, accounting for approximately 70% of the known and tested drug targets [3]. Therefore, study of the membrane protein structure and function is currently a popular topic in the chemistry and biology fields.

Over the past few decades, many protein structures have been determined. However, most of them are globular proteins, while only a few membrane proteins have been structurally determined. In fact, in the Protein Data Bank (PDB), less than 1% of the solved structures are membrane proteins [4]. This is not because of their less importance than globular proteins, but is mainly due to the technical challenges imposed on the expression of membrane proteins in large quantities, dissolution from the biomembrane, and crystallization [5].

Considering this situation, it is desirable to predict membrane protein structures by developing computational methods. For globular proteins, a plethora of different methods have been developed to predict their structures [6]–[8], serving as a useful reference for membrane protein structure prediction. Previous work suggests that residue contact prediction with an accuracy higher than 22% is helpful for *ab initio* simulation of globular protein structures [9]. It is likely that this observation might also apply to membrane protein structure prediction. A recent study suggests that even if very limited experimental information with regards to residue-residue contacts is known, a model within 4 Å of the native structure can still be attained [10]. Thus, the predicted residue contact pairs and interacting helices in membrane proteins could act as useful structural constraints in membrane protein structure simulation and prediction.

With respect to globular proteins, a variety of computational methods have been developed to predict residue contact pairs [11]–[23]. These methods can be further categorized into four types: 1) correlated mutation-based residue contact identification [13], [15]–[17]; 2) machine learning methods, including support vector machines (SVMs), neural networks and Markov models [11], [12], [14], [18]–[20]; 3) structural template-based approaches [21]–[23]; 4) the combination of the first and second type to predict residue contact pairs [11], [12]. More details about the four types of computational methods can be found in Wu and Zhang's work [9].

In contrast, fewer methods exist to predict residue contacts particularly in membrane proteins. So far, there are mainly three methods to predict residue contact pairs in alpha-helical transmembrane (TM) proteins, which are TMHcon [24] using artificial neural network, TMhit [25] using SVM, and MEMPACK [26] using SVM. All of the three methods first predict residue contact pairs between different TM helices and then infer helix-helix interactions based on the predicted residue contact pairs.

In this article, we adopted two different definitions of residue contacts and applied a random forest (RF) algorithm to solve the challenging task of predicting residue-residue contact and helix-helix interaction in alpha-helical TM proteins. We termed our predictor as TMhhcp (TM helix-helix contact predictor). As a result, our approach achieved the top *L*/5 residue contact prediction accuracies of 49.5% and 48.8%, based on the two different definitions of residue contacts, respectively, providing better performance than TMHcon and MEMPACK. We further utilized the predicted residue contacts to identify interacting helical pairs and attained the Matthew's correlation coefficients (MCCs) of 0.430 and 0.424, respectively. Moreover, we also performed feature selection experiments to evaluate and select important informative features contributing to performance improvement.

## Methods

### Datasets

In this work, we used a well-prepared training set containing 62 TM proteins that were previously used by TMHcon [24]. This training dataset was compiled from PDBTM [27] (version of September 17,2007), which contained 677 alpha-helical TM structures, and the dataset provided by the Stephen White laboratory (http://blanco.biomol.uci.edu/Membrane_proteins_xtal.html; version of September 17, 2007). Briefly, all the solved protein structures in this dataset have a resolution better than 3.5 Å, with a pair-wise sequence identity of less than 40%. The topology data were obtained from TOPDB [28], with the exception of two protein structures 2UUH (chain: A) and 1ORQ (chain: C). Their TM positions were extracted from PDBTM [27] and the corresponding orientations were obtained from OPM [29].

In addition, we created a test set in order to validate the prediction performance of our method. First, we downloaded the alpha-helical TM protein chains from a newer version of PDBTM (October 1, 2010), which contained 1,070 alpha-helical TM proteins, and selected those having at least three TM helices as well as sharing less than 40% sequence identity to protein chains in the training set. We then submitted the PDB IDs of these protein chains to the PISCES server [30] that returned a non-redundant PDB ID list. This was used as the test set. Finally, this test set contained 21 TM protein chains whose structures were all determined by X-ray diffraction with a resolution less than 3 Å and had a pair-wise sequence identity less than 40%. The TM segments of proteins in the test set were derived from PDBTM [27] and the corresponding orientations were obtained from OPM [29].

### Definitions of residue contacts

Existing residue contact predictors for TM proteins have used different definitions of residue contacts and helix-helix interactions. TMHcon [24] defined that two residues within different TM helices were in contact, if the minimal distance between the heavy atoms of the side chain or backbone was less than 5.5 Å. Two TM helices were interacting with each other if they had at least one contact residue pair. The definition proposed by TMHcon is denoted as DEF1 in this study. The second definition was given by MEMPACK [26]. It required the C-beta atom (C-alpha atom in the case of glycine) to have a distance <8 Å for two residues to be in contact and at least one residue contact pair to be present for two helices to be interacting. The definition proposed by MEMPACK is denoted as DEF2. More recently, Duarte *et al.* pointed out that a distance cut-off of 9 to 11 Å around the C-beta atoms could represent the 3D structure most accurately when applied to the contact maps [31]. In this study, we constructed two different types of RF predictors and evaluated their prediction performance based on the two different contact definitions.

### Input features

**Figure 1:**
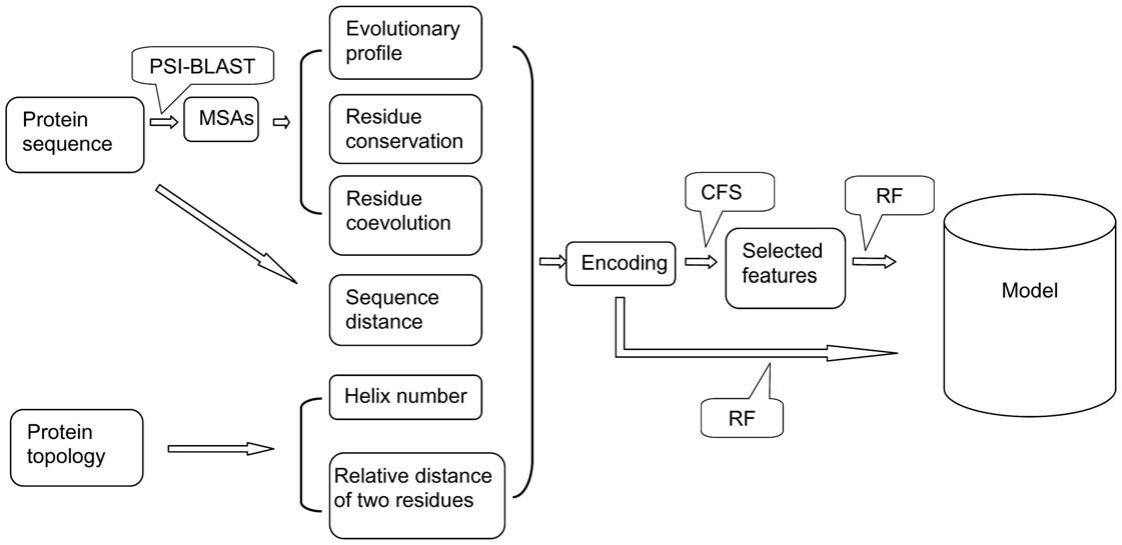
Schematic overview of RF-based model building process of the TMhhcp approach.

We used the RF algorithm [32] to predict residue contacts between different TM helices. The schematic overview of our TMhhcp approach is depicted in Figure 1. The model building process consists of three major steps: feature extraction and encoding, feature selection, and model building (Figure 1). In order to build the RF-based prediction models, several different types of features were extracted and used as input to train the models. In the subsequent sections, we will describe in more detail individual input features, feature selection and model building procedures.

#### Evolutionary profile

The evolutionary profile, represented by a Position-Specific Scoring Matrix (PSSM), was first constructed by running PSI-BLAST [33] search against the UNIREF90 database with three iterations and an e-value cut-off of 1e-10. In a PSSM, each residue was represented by a 20-dimensional vector, denoting the frequencies of the 20 amino acids appearing at the corresponding position in the PSSM. For a residue of interest, a nine-residue sliding window centered on that residue was used to extract its evolutionary profile. If there were less than four residues on one side of a residue, each non-existing position was represented by a 20-dimensional zero-valued vector. Finally, a residue pair was encoded by a 360-dimensional vector.

#### Residue coevolution

Three correlated mutation methods, MIc [16], OMES [34] and McBASC [35] were used to infer the coevolving residue pairs from multiple sequence alignments (MSAs). The MSAs were first obtained through the PSI-BLAST search against UNIREF90 as described above. Then, the MSAs were filtered based on the following criteria: 1) In an MSA, residue columns that do not belong to any TM segment were removed. 2) Any sequence in the MSA containing ≥25% gaps in any TM segment was also removed. 3) The MSA was further filtered to ensure that the sequence identity between any two sequences was ≤90% (It should be noted that the sequence identity here was based merely on all TM segments rather than the whole sequence). Moreover, the calculated correlated mutation scores by the three methods were standardized using the formula 
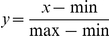
, where min and max are the minimal and maximal correlated mutation values in the query sequence. For a residue pair *ij*, we encoded its coevolutionary feature as an input vector: (*S_i_*_−4, *j*−4_, *S_i_*_−3, *j*−3_, …, *S_i_*_+3, *j*+3_, *S_i_*_+4, *j*+4_), where *S_i_*_, *j*_ represents the correlated mutation score for residue pair *ij*. The encoded sequence segment (*i*−4, …, *i*+4 and *j*−4, …, *j*+4) is in the orientation from the cytoplasm to the extracellular side of the membrane. Thus, a 27-dimensional vector was obtained from the residue coevolution encoding scheme.

#### Residue conservation

The sequence conservation score of a residue position, generally considered to be closely correlated with the burial status of the residue [36]–[39], was calculated according to the Shannon's entropy at this position in an MSA. The conservation scores were also standardized as above. Similarly, for a residue of interest, a nine-residue sliding window centered on that residue was employed to extract its conservation profile. For a residue pair of interest, the residue conservation encoding scheme resulted in an 18-dimensional feature vector.

#### Relative distance of two residues within TM helices

Suppose that there are two residues residing at positions *p*_1_ and *p*_2_ in two different TM helices with lengths *l*_1_ and *l*_2_, respectively. Their relative distance from each other within TM helices can be calculated as |*p*_1_/*l*_1_−*p*_2_/*l*_2_|. It should be noted that the residue position 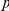 ranges from 1 to 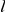 within a TM helix that has 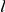 residues from the cytoplasm to the extracellular side of the membrane. This feature stands for the distance of two residues perpendicular to the surface of the membrane.

#### Other types of features

Other features collected for each residue pair and used by TMhhcp predictors include the residue distance in the primary sequence and the number of TM helices.

### Feature selection

The aforementioned feature construction resulted in a 408-dimensional feature vector. In order to create a more condensed model with less noisy and uninformative features, we need to perform feature selection experiments to select the most meaningful features. For this purpose, we used the correlation-based feature selection (CFS) [40] to select a subset of features that, individually, have a higher ability of predicting the class but have little inter-correlation. The correlation between features *X* and Y can be measured using the following function:
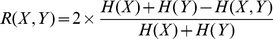
(1)where *H* is the Shannon's entropy of the feature. The appropriateness of a set of features is determined using
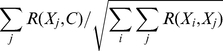
(2)where *C* is the class and the indices *i* and *j* iterate over all features of the set. In order to avoid exhaustive search of all feature subsets, a confined search method called BestFirst [41] was used. In our work, we randomly selected 10 sets of samples with equal numbers of positive and negative samples. 10 feature subsets were then obtained and integrated into a feature set that was used to train the RF classifier. The feature selection process was performed using the Weka package [42]. The final selected features and their scores are listed in the supplementary material.

### Model building

According to the residue contact definitions, residue pairs in the training set are classified into contacts (positive samples) and non-contacts (negative samples). In principle, the training set should include as many residue pairs as possible. For a membrane protein structure, however, the number of non-contact residue pairs is considerably higher than that of contact pairs, leading to the problem of imbalance [43]. Including all the non-contact residue pairs into the training set would end up with a long training time and fewer correct predictions of contact residue pairs. To overcome this issue, we included all the contact residue pairs and randomly selected non-contact residue pairs with the ratio of 1∶4 contact pairs in the training set, as suggested previously [9].

The RF algorithm [32] is a popular machine learning method that has been used in diverse bioinformatics studies with excellent performances [44]–[46]. It grows many classification trees and chooses the classification with the most votes from all the trees. Each tree is grown as follows: for a training set of *N* cases and *m* variables, sample *N* cases with replacement from the original data to grow the tree. A number *m*≪*M* is specified such that at each node *m* variables are selected randomly to best split the nodes. Each tree is grown as large as possible. The error of RF depends on the strength of each individual tree and the correlation between them.

In our work, we built RF models based on all the features and the selected features through CFS respectively to examine the effectiveness of the feature selection. We generated 100 trees for each model and set *m* to the default value of 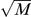, because we found that there was no significant difference in the resultant prediction performances at different adjusted *m* values. The RF algorithm was implemented using the randomForest R package [47].

### Evaluation measures

In order to assess the performance of the RF-based predictors, we performed strict jackknife cross-validation tests, i.e. all residue pairs in a singled-out sequence were predicted and tested using the model trained using all the other residue pairs of the remaining sequences in the training set. In addition, we also tested our method on an independent test set. It is noteworthy that the performance was assessed at the whole protein chain level and as a result, the overall performance was obtained by averaging the individual prediction results of all the tested protein chains.

For prediction of residue contact pairs, the top *L*/5 predictions were ranked as the residue contact pairs, where *L* is the sum of lengths for all TM segments of a protein chain. The accuracy, defined as the number of correctly predicted residue contacts divided by the number of predicted residue contacts (i.e. *L*/5), was used to assess the prediction performance. The top *L*/5 prediction accuracy has been consistently used to evaluate the prediction performance of the developed predictors [26]. Moreover, we also presented the accuracies for the top *L*/2 and *L* predictions in this study in order to comprehensively evaluate the performance of the RF-based predictors. The coverage (percentage of correctly predicted contacts out of the observed contacts) was also computed. We used “δ-analysis” to investigate the fraction of correctly predicted contacts within an interval of δ around the observed contacts [24], [25]. We set δ = 4 to determine the percentage of predicted contacts about one turn around the observed contacts. We also drew the precision-recall curves to show the precision (i.e. the accuracy in this study) as the coverage increases. For helix-helix interaction prediction, accuracy (percentage of correctly predicted interactions out of predicted interactions), sensitivity, specificity and MCC [48] were calculated.

## Results and Discussion

### Model building

The RF approach has been used in previous studies and has demonstrated excellent prediction performance. In this study, we describe its first application to predict residue-residue contacts and helix-helix interactions in TM proteins. Our approach was termed as TMhhcp and four different TMhhcp predictors were constructed. TMhhcp1 and TMhhcp2 are two RF predictors according to DEF1 and DEF2, respectively. These were built based on the training dataset with all features, while TMhhcp_cfs1 and TMhhcp_cfs2 are another two RF predictors based on the selected features using the CFS approach according to DEF1 and DEF2, respectively.

### Prediction performance evaluated based on the jackknife cross-validation tests

We performed leave-one-protein-out jackknife cross-validation tests to assess the prediction performance of our method. The prediction performances are presented in Table 1. We can see that the prediction accuracies obtained by the models based on all features (TMhhcp1 and TMhhcp2 predictors) are higher than those of the models based on the selected features (TMhhcp_cfs1 and TMhhcp_cfs2 predictors). However, for the δ-analysis, the two types of RF models provide comparable performances. This suggests that they have similar abilities in predicting residue contacts localized within a sequence separation of one helix turn of observed contacts.

**Table 1:** Prediction performance comparison of different methods based on the jackknife cross-validation test.

Predictor	Accuracy (%)	Coverage (%)	Accuracy (δ = 4) (%)
TMhhcp1^a^	49.5	8.2	83.9
TMhhcp_cfs1^a^	45.8	7.4	83.8
TMhhcp2^b^	48.8	8.4	83.7
TMhhcp_cfs2^b^	46.6	8.0	82.4
TMHcon^a^	25.9	3.5	78.5

a Residue contact definition 1 (i.e. DEF1).

b Residue contact definition 2 (i.e. DEF2).

Since we used the same dataset of protein chains and evaluation measures as TMHcon, we directly compared our prediction results with TMHcon. As shown in Table 1, TMhhcp1 achieved a much higher accuracy than TMHcon. A possible reason might be that we have included more instances of non-contact residue pairs into the training set. Even using a 1∶1 ratio of contacts to non-contacts, the accuracy of our method still reached 43.3%. Thus, the favorable performance of our method may be attributed to the application of the RF method and the different features we used to build the predictors.

We calculated the top *L*/5 predictions of our method and compared the results with TMHcon. In addition, we also calculated the top *L*/2 and *L* predictions of our method (Table 2), as these two values were frequently used by other researchers in this field. It could be seen that for the top *L*/2 predictions, TMhhcp1 and TMhhcp2's accuracies were 42.8% and 43.0% respectively, while for the top *L* predictions, TMhhcp1 and TMhhcp2's accuracies were 34.6% and 35.1%, respectively.

**Table 2:** Jackknife cross-validation performance of different TMhhcp models in terms of the top *L*/2 and *L* predictions.

	Top *L*/2 predictions			Top *L* predictions		
Predictor	Accuracy (%)	Coverage (%)	Accuracy (δ = 4) (%)	Accuracy (%)	Coverage (%)	Accuracy (δ = 4) (%)
TMhhcp1^a^	42.8	17.4	81.8	34.6	27.6	77.9
TMhhcp_cfs1^a^	37.5	15.0	79.3	30.2	24.0	76.4
TMhhcp2^b^	43.0	18.3	80.9	35.1	29.1	76.8
TMhhcp_cfs2^b^	40.1	17.0	79.5	32.3	26.8	75.9

a Residue contact definition 1 (i.e. DEF1).

b Residue contact definition 2 (i.e. DEF2).

**Figure 2:**
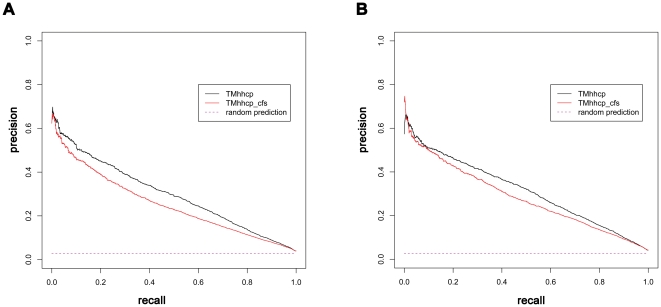
The precision-recall curves based on the jackknife cross-validation tests. Panels A and B were generated based on DEF1 and DEF2, respectively. The precision-recall curve analysis was conducted at the whole protein chain level, and the precision-recall curves in panels A and B reflected the average precision-recall curves for the 62 tested protein chains. The average ratios of contact residue pairs to the total residue pairs were 0.028 and 0.027, according to DEF1 and DEF2, respectively. Therefore, the corresponding random prediction precision-recall curves in panel A and B were horizontal lines with the precision value of 0.028 and 0.027, respectively.

To assess the average performance of the TMhhcp models on the 62 transmembrane protein chains, we referred to Algorithm 3 of Tom Fawcett's work [49] to draw the corresponding precision-recall curves (Figure 2). Note that the precision-recall curve analysis was conducted at the whole protein chain level. Firstly, the precision-recall curve of each tested protein chain was prepared. Then, the average precision-recall curve (Figure 2) was generated by plotting the average precision values of the 62 tested proteins at different recall controls. The area under the precision-recall curve (AUPRC) was further used to quantify the performance. Generally, a higher AUPRC value corresponds to a better performance. According to DEF1, the AUPRC values for TMhhcp and TMhhcp_cfs were 0.300 and 0.254, respectively. According to DEF2, the AUPRC values for TMhhcp and TMhhcp_cfs were 0.314 and 0.283, respectively.

### Prediction performance evaluated on the independent test set

In order to further validate our method, we also performed an independent test to compare the prediction performance of our method with two other methods (TMHcon and MEMPACK). As shown in Table 3, the results obtained by our method were as accurate as those in the jackknife cross-validation tests (Table 1). The accuracies for TMhhcp1, TMhhcp_cfs1, TMhhcp2 and TMhhcp_cfs2 were 48.1%, 48.6%, 47.3% and 46.5%, respectively. In the case of the δ-analysis, the accuracies reached 84.4%, 81.8%, 82.8% and 79.5%, respectively. Moreover, TMhhcp models consistently provided relatively good performance for the top *L*/2 and *L* predictions on the independent test set (Table 4).

**Table 3:** Prediction performance of different methods evaluated on the independent test set.

Predictor	Accuracy (%)	Coverage (%)	Accuracy (δ = 4) (%)
TMhhcp1^a^	48.1	6.1	84.4
TMhhcp_cfs1^a^	48.6	6.1	81.8
TMhhcp2^b^	47.3	5.9	82.8
TMhhcp_cfs2^b^	46.5	5.9	79.5
TMHcon^a^	23.6	3.0	83.4
MEMPACK1^a^	36.2	10.4	63.0
MEMPACK2^b^	34.6	17.4	61.0

a Residue contact definition 1 (i.e. DEF1).

b Residue contact definition 2 (i.e. DEF2).

**Table 4:** Performance comparison of different TMhhcp models for the top *L*/2 and *L* predictions evaluated on the independent test set.

	Top *L*/2 predictions			Top *L* predictions		
Predictor	Accuracy (%)	Coverage (%)	Accuracy (δ = 4) (%)	Accuracy (%)	Coverage (%)	Accuracy (δ = 4) (%)
TMhhcp1^a^	40.4	12.8	81.8	35.0	22.3	78.5
TMhhcp_cfs1^a^	39.6	12.4	79.3	32.5	20.3	77.4
TMhhcp2^b^	40.7	12.9	79.8	35.1	22.5	77.9
TMhhcp_cfs2^b^	39.0	12.3	79.4	31.9	20.2	76.2

a Residue contact definition 1 (i.e. DEF1).

b Residue contact definition 2 (i.e. DEF2).

To benchmark the performance of TMHcon and MEMPACK on the independent test set, the corresponding stand-alone versions of TMHcon and MEMPACK were downloaded and installed in our local machine and the independent test set was processed. As can be seen from Table 3, our method still outperformed TMHcon when evaluated on this independent test set.

In contrast, MEMPACK, another helix-helix interaction predictor, used a different class decision mode by directly predicting residue contacts based on the score generated by an SVM predictor. We found that the classification mode used by MEMPACK led to biased prediction results, possibly due to the small ratio of contacts to non-contacts in proteins. In particular, there were four protein chains in the independent test set for which MEMPACK failed to predict any residue contact. The precision values for the four protein chains were set to 0. Moreover, there is a possibility that the independent test set we used might have contained homologous sequences that had higher sequence identity with those in the training set of MEMPACK. However, despite this possibility, our TMhhcp predictors outperformed MEMPACK on the independent test set and achieved higher accuracies of 48.1% and 47.3%, compared to 36.2% and 34.6% of MEMPACK based on two different definitions DEF1 and DEF2, respectively. In addition, we compared the performance of TMhhcp and MEMPACK, when the four protein chains were excluded, as shown in Table S1. TMhhcp achieved the accuracies of 53.1% and 51.1%, while MEMPACK achieved the accuracies of 44.7% and 42.7%, based on the two definitions of DEF1 and DEF2, respectively. The performance of TMhhcp for the four protein chains is given in Table S2. It should be noted that the average coverage of MEMPACK was much larger than that of TMhhcp. This is because MEMPACK predicted many more residue contacts for some proteins, possibly due to its different classification mode. As a result, the average coverage of MEMPACK was comparatively large. Nevertheless, for the top *L*/5 classification mode, the number of predicted residue contacts was fixed.

**Figure 3:**
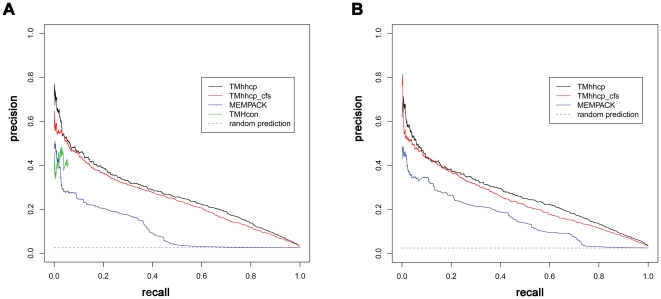
The precision-recall curves based on the independent test set. Panels A and B were generated based on DEF1 and DEF2, respectively. The precision-recall curve analysis was conducted at the whole protein chain level, and the precision-recall curves in panels A and B reflected the average precision-recall curves for the 21 tested protein chains. According to DEF1 or DEF2, the average ratio of contact residue pairs to the total residue pairs on the independent test set was 0.025. Therefore, the corresponding random prediction precision-recall curve in panel A or B was a horizontal line with the precision value of 0.025.

A fair and better way to evaluate and compare the performance of different predictors might be the precision-recall curves. Similar to the generation of precision-recall curves for the jackknife cross-validation test, the precision-recall curves of different predictors based on this independent test set were given in Figure 3. The corresponding AUPRC values for TMhhcp, TMhhcp_cfs and MEMPACK were 0.268, 0.249 and 0.107, respectively, according to DEF1. The AUPRC values for TMhhcp, TMhhcp_cfs and MEMPACK were 0.265, 0.242 and 0.156, respectively, according to DEF2. However, as TMHcon and MEMPACK were developed for specific purposes, the plotted precision-recall curves could not reflect their performance across a wide range of varying thresholds. For instance, TMHcon was developed to provide the scores for the top *L*/5 predicted contacts. Thus, only a portion of its curve under lower recall values could be plotted and its corresponding AUPRC value under the complete curve could not be calculated. In the case of MEMPACK, it predicted residue contacts on the residue level rather than the protein level and its output scores for predicted non-contacts were simply set as zero. As a result, when drawing the curve, residue contacts that scored as zero were ranked randomly and the precision-recall curve of MEMPACK at higher recall values was close to a random prediction (Figure 3). From a practical perspective, more attention should be paid to the performance at higher precision values. Although the complete precision-recall curves of TMHcon and MEMPACK were not plotted, the performance comparison among TMhhcp, TMHcon and MEMPACK at higher precision values can be fairly benchmarked. From Figure 3, we can see that TMhhcp models achieved higher recall values than TMHcon and MEMPACK at a precision control of 40%. In addition, when only considering protein chains for which MEMPACK predicted at least one residue contact, the corresponding precision-recall curves of TMhhcp and MEMPACK are given in Figure S1. Again, Figure S1 shows that TMhhcp outperformed TMhhcp. In summary, the precision-recall curves suggest that our method has outperformed TMHcon and MEMPACK for residue-residue contact prediction based on this independent test set.

### Important informative features

We further carried out feature selection experiments in order to select the most meaningful features and obtain a concise model. As a result, three significant features with the highest scores were obtained: relative distance of two residues within two TM helices, residue separation in the primary sequence and the correlated mutation score calculated by the MIc method [16]. It is reasonable to think that two residues separated within a small degree on the Z-axis tend to contact with each other. Therefore, the relative distance of two residues within two helices is reasonably an important feature for performance improvement.

**Figure 4:**
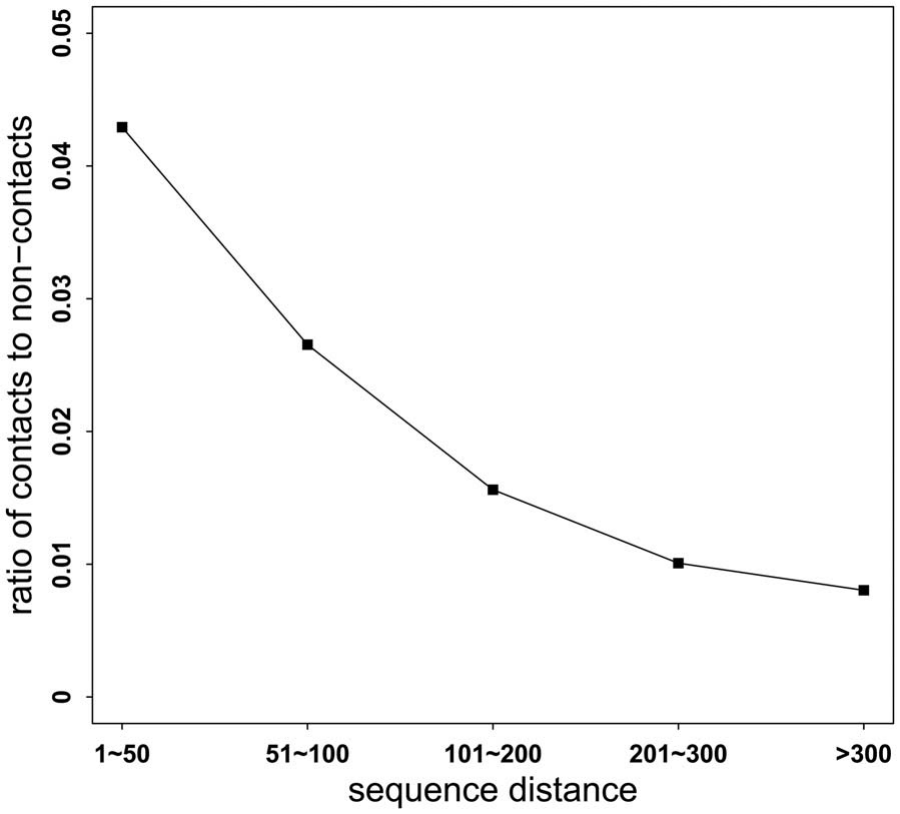
The ratio of contacts to non-contacts according to sequence distance. This figure describes the ratio of contacts to non-contacts according to the grouping of their sequence distance based on DEF1.

In addition, residue separation in the primary sequence is another important feature. To assess the impact of sequence separation distance on residue contact prediction, we compared the ratio of contacts to non-contacts, which was grouped according to the grouping of their sequence separation distance based on DEF1 (Figure 4). With the increase of sequence separation distance, the ratio of contacts to non-contacts decreases. This suggests that in the folding process of TM proteins, residue contacts prefer to occur among those separated by short sequence distances. We also calculated the ratio of contacts to non-contacts based on DEF2 and observed similar trends.

**Figure 5:**
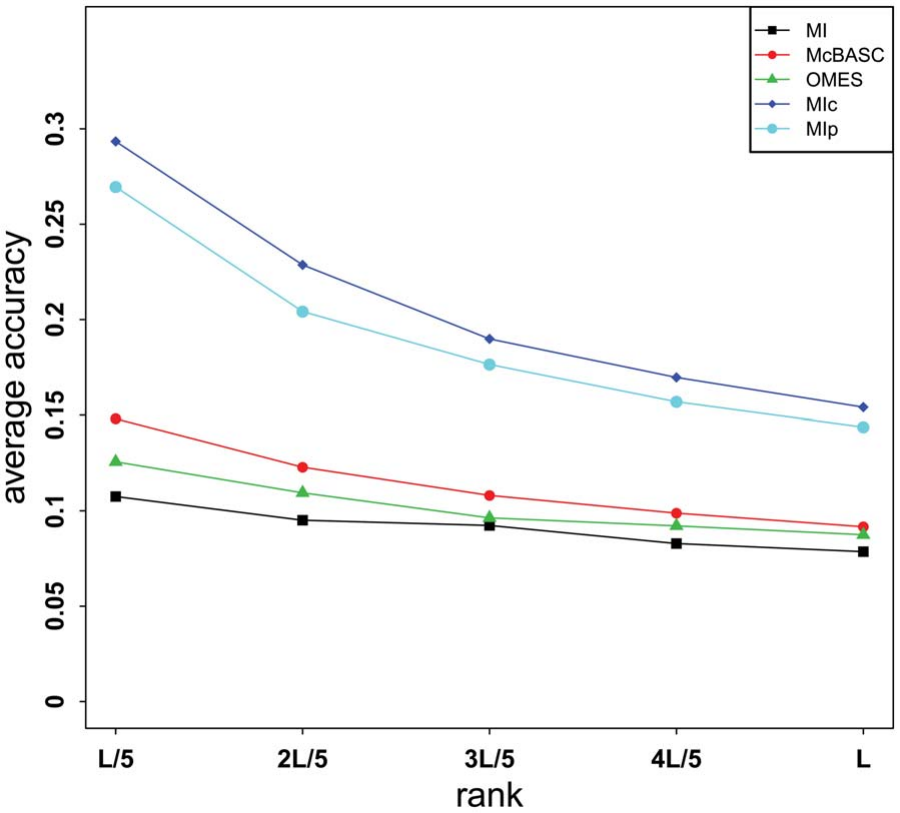
The average prediction accuracy of five covariance algorithms. This figure gives the average prediction accuracy of five different covariance algorithms to predict residue contacts on the training set using DEF1. *L* is the sum of lengths of all TM segments of a protein chain.

Correlated mutations have been previously used to predict residue contacts [50] based on the observation that interacting residues have tendency to coevolve [51]. Recently, a new correlated mutation algorithm called MIp [15] that removes the influence of phylogeny or entropy can significantly improve the prediction accuracy of residue contacts. Following the idea of MIp, an improved measure called MIc [16] was further proposed to calculate the covariance of two residues, with demonstrated performance better than the MIp score. In our work, we found that both MIp and MIc scores produced similar results in predicting residue contacts for TM proteins, but MIc achieved slightly better prediction accuracy. We then incorporated the MIc score along with the coevolutionary scores generated by another two commonly used algorithms, i.e. OMES [34] and McBASC [35], into our feature set. Among them, the MIc score was retained as one of the three most important features after feature selection. To provide a comprehensive assessment of different covariance algorithms, we tested the performances of McBASC, OMES, MI [17], MIp and MIc on the training set using DEF1 (Figure 5). When the number of predicted contacts was fixed at different ratios to the protein chain's length, the accuracy for each algorithm increased in the order: MI<OMES<McBASC<MIp<MIc. Because MI performed worst in residue contact prediction, we did not incorporate it into the feature set of TMhhcp. Similarly, using DEF2, the above five covariance algorithms led to the same conclusion. When ranking the *L*/5 highest scoring residue pairs as the predicted contact pairs, it is worth mentioning that the average accuracy of MIc was 29.3%, which is even higher than that of TMHcon (25.9%) (see Figure 5 and Table 1).

In addition, the evolutionary profile in the form of PSSM is also an important feature, because it represents the evolutionary information of a protein sequence and constitutes the majority of the selected features. Table S3 and S4 in the Supporting Information list the selected features based on two different residue contact definitions DEF1 and DEF 2, respectively.

### Application to helix-helix interaction prediction

An important application of residue contact prediction for TM proteins is to predict helix-helix interactions, namely, the interacting helical pairs. According to the prediction rules of TMHcon and MEMPACK, two TM helices were predicted to be interacting if they have at least one predicted residue contact pair. Based on this definition, we used the top *L*/5 predicted residue contacts by our TMhhcp predictors to predict helix-helix interactions and compared the prediction performance with other methods based on the independent test set. Initially, the prediction models of TMhhcp were built by using all features to make the prediction of interacting helical pairs. However, it turned out that the prediction accuracy in this way was lower than the TMhhcp models that were built using the selected features only. The prediction performance of these two types of TMhhcp models is displayed in Table 5. It can be seen that using DEF1, both TMhhcp_cfs1 and MEMPACK1 achieved the highest accuracy of 80.4%. Nevertheless, TMhhcp_cfs1 correctly predicted more interacting helical pairs than TMHcon and MEMPACK1 with higher sensitivity values. On the other hand, using DEF2, TMhhcp_cfs2 attained the highest accuracy, and predicted more interacting helical pairs than MEMPACK2 with higher sensitivity (Table 5). We also calculated the MCC measures of all the TMhhcp models, which were all higher than 0.4 (Table 5). As a comparison, the MCC values of TMHcon, MEMPACK1 and MEMPACK2 were 0.322, 0.278 and 0.287, respectively, which are much lower than TMhhcp models. Altogether, these results suggest that our TMhhcp models clearly outperformed the other two methods in the task of predicting interacting helical pairs.

**Table 5:** Prediction performance of helix-helix interaction on the independent test set.

Predictor	Accuracy (%)	Sensitivity (%)	Specificity (%)	MCC
TMhhcp1^a^	79.1	54.5	86.2	0.430
TMhhcp_cfs1^a^	80.4	53.7	88.9	0.435
TMhhcp2^b^	77.5	50.8	88.0	0.424
TMhhcp_cfs2^b^	79.3	45.2	90.4	0.407
TMHcon^a^	76.7	39.5	88.5	0.322
MEMPACK1^a^	80.4	27.0	93.7	0.278
MEMPACK2^b^	76.1	29.2	92.6	0.287

a Residue contact definition 1 (i.e. DEF1).

b Residue contact definition 2 (i.e. DEF2).

### Overlap of the predictions of different tools represented by Venn diagrams

**Figure 6:**
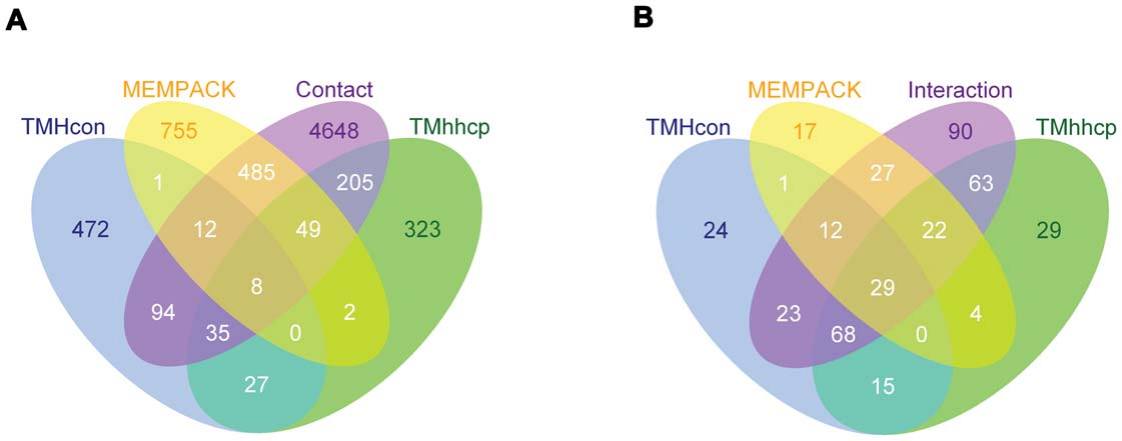
Two Venn diagrams for the predicted residue contacts and helix-helix interactions by three predictors. The two Venn diagrams display the complementation between the three predictors, TMHcon, MEMPACK and TMhhcp, to predict residue contacts and helix-helix interactions. The corresponding residue contact definition is based on DEF1. ‘Contact’ in panel A represents the observed residue contacts of protein chains in the test set, while ‘Interaction’ in panel B denotes the observed helix-helix interactions in the test set.

In order to analyze the overlap of the predictions of the three predictors TMHcon, MEMPACK and TMhhcp based on DEF1, we generated the Venn diagrams based on their prediction results (See Figure 6A and 6B for the predicted residue contacts and interacting helical pairs, respectively). From the Venn diagrams, accuracies and sensitivities, listed in Table 3 and Table 5, can be easily calculated. For residue contact prediction, the overlap of the predicted residue contacts for every two predictors accounted for less than 11% of their own predictions (Figure 6A). This suggests that the three prediction methods are strongly complementary with each other. For helix-helix interaction prediction, the overlap of the predicted interacting helical pairs for every two predictors accounted for less than 66% of their own predicted interacting helical pairs (Figure 6B), suggesting that the three prediction methods are complementary with each other to some extent.

### Case studies

**Figure 7:**
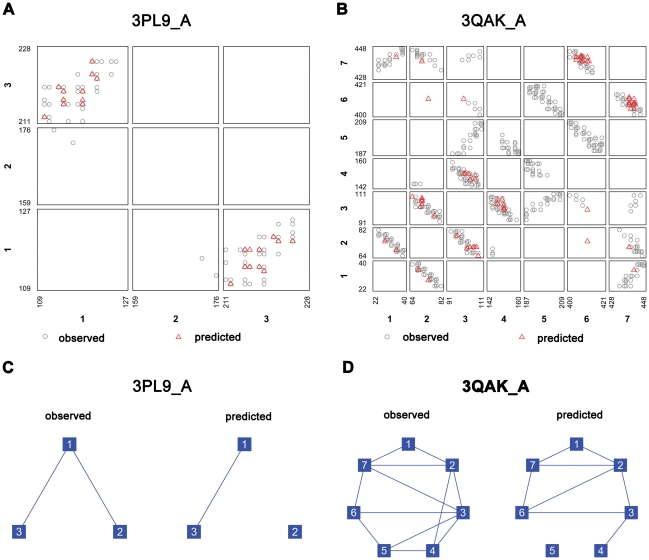
Case studies. This figure displays the performance of TMhhcp on two recently structure solved TM proteins, the Spinach minor light-harvesting complex CP29 (PDB ID: 3PL9, chain: A) and the human adenosine A2A receptor bound with agonist (UK-432097) (PDB ID: 3QAK, chain: A). Panels A and B plot the observed and predicted residue contacts of 3PL9_A and 3QAK_A, respectively. Each grid contains the residue contacts of the corresponding two TM segments. The edges of a grid represent the lengths of the corresponding two TM segments. Panels C and D give the observed and predicted interacting helical pairs of 3PL9_A and 3QAK_A, respectively, where the two boxes connected by a line represent an interacting helical pair.

In order to test the performance of TMhhcp under “real-life” conditions, we applied TMhhcp using DEF1 to two recently solved TM proteins: the Spinach minor light-harvesting complex CP29 (PDB ID: 3PL9, chain: A) [52] and the human adenosine A2A receptor bound with agonist (UK-432097) (PDB ID: 3QAK, chain: A) [53]. The maximal sequence identities of the two protein chains to those used in the training set are 41.2% and 27.0%, respectively. Regarding the top *L*/5 predictions, TMhhcp achieved the residue contact accuracies of 90.9% and 41.4% for the two protein chains, respectively. The accuracies for δ-analysis are 100% and 96.6%, respectively, when the top *L*/5 predictions were predicted as residue contacts. These satisfying results suggest that TMhhcp is a powerful tool in predicting residue contacts in TM proteins and performs extremely well at predicting residue contacts within one helix turn of the observed contacts (Figure 7 A and 7B). Furthermore, for helix-helix interaction prediction that requires at least one predicted residue contact pair, the predicted helix-helix interaction pattern formed by the predicted interacting helical pairs clearly resembled the corresponding observed pattern (Figure 7C and 7D). For instance, seven out of the twelve observed interacting helical pairs in 3QAK_A were correctly predicted (Figure 7D).

### The TMhhcp web server

In order to provide a public service of TM protein residue contact and helix-helix interaction prediction, a web server called TMhhcp has been developed and made freely available at http://protein.cau.edu.cn/tmhhcp. At the prediction webpage, the user is required to input the query sequence and its topology. We provided two models to predict residue contacts of TM proteins according to the two different residue contact definitions. The topology of the query sequence should be described as a sequence consisting of “H”, “I”, “O” and “U” that represent TM segment, inside position, outside position and unknown topology, respectively. To obtain the topology information, the users may need to employ some well-established TM topology predictors such as TMHMM [54] and HMMTOP [55]. To facilitate the method developers, the training data and test data used in this work are also downloadable at the help webpage of TMhhcp. Currently, a four-CPU DELL Linux system with 16 GB of main memory hosts the TMhhcp web server. The computational time is mainly decided by the PSI-BLAST search and the covariance algorithm McBASC. For instance, it costs approximately five minutes to finish the prediction of 3QAK_A that contains 488 residues and 7 TM helices.

### Conclusions

In this study, we applied the RF algorithm to predict residue-residue contacts in TM proteins and achieved better performance than two state-of-the-art methods TMHcon and MEMPACK. We performed feature selection to select the most meaningful features and analyzed the selected features that contribute to the improved performance for predicting residue contacts. We found that prediction of residue contacts can be significantly improved using the descriptors of the relative distance of two residues of interest and their sequence separation in the primary sequence. In addition, the correlated mutation score, as a third important feature, has important impact on residue contact prediction. It has also been established that our method outperformed two existing methods TMHcon and MEMPACK for predicting helix-helix interactions of TM proteins. We hope our method will become a valuable tool for predicting the structural properties of TM proteins and can help to gain useful insights into their structure and function.

## Supporting Information

Figure S1 **The precision-recall curves of MEMPACK and TMhhcp based on 17 tested protein chains in the independent test.** Panels A and B were generated based on DEF1 and DEF2, respectively. The precision-recall curves reflected the average precision-recall curves for the 17 tested protein chains for which MEMPACK predicted at least one residue contact. predicted at least one residue contact.
(TIF)

Table S1 **Prediction performance of TMhhcp on the 4 protein chains for which MEMPACK failed to predict any residue contact.**
(DOC)

Table S2 **Prediction performance of TMhhcp on the 4 protein chains for which MEMPACK failed to predict any residue contact.**
(DOC)

Table S3 **The selected features based on the residue contact definition DEF1.**
(DOC)

Table S4 **The selected features based on the residue contact definition DEF2.**
(DOC)
