# Supplementary figures and images for: Predicting Residue-Residue Contacts and Helix-Helix Interactions in Transmembrane Proteins Using an Integrative Feature-Based Random Forest Approach
Source: PLoS One. 2011 Oct 28;6(10):e26767. doi: 10.1371/journal.pone.0026767 (PMC3203928; doi:10.1371/journal.pone.0026767)

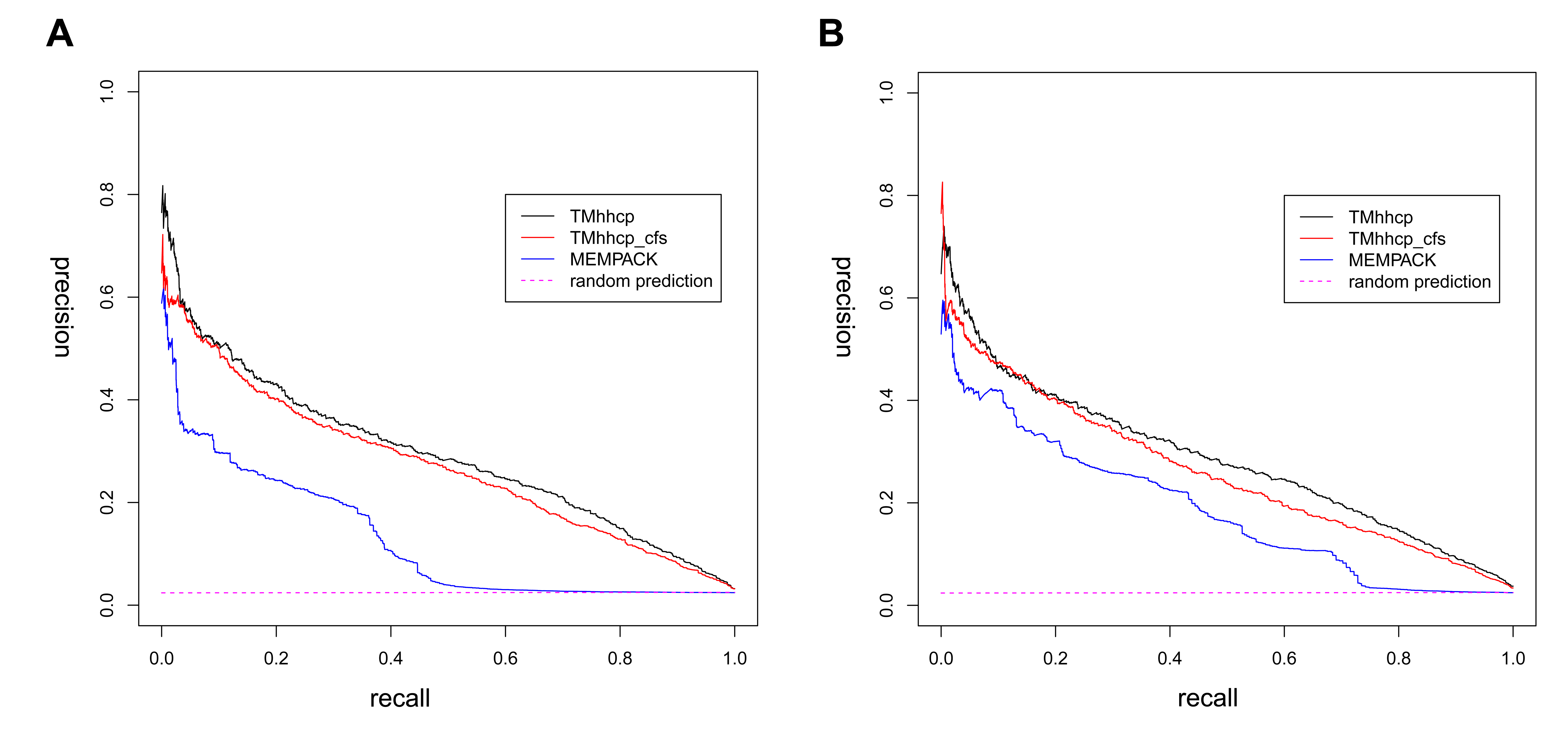

Supplement: Figure S1 — The precision-recall curves of MEMPACK and TMhhcp based on 17 tested protein chains in the independent test. Panels A and B were generated based on DEF1 and DEF2, respectively. The precision-recall curves reflected the average precision-recall curves for the 17 tested protein chains for which MEMPACK predicted at least one residue contact. predicted at least one residue contact. (TIF) [file pone.0026767.s001.tif]
