# Supplementary material for: Predicting Residue-Residue Contacts and Helix-Helix Interactions in Transmembrane Proteins Using an Integrative Feature-Based Random Forest Approach
Source: PLoS One. 2011 Oct 28;6(10):e26767. doi: 10.1371/journal.pone.0026767 (PMC3203928; doi:10.1371/journal.pone.0026767)
Supplement: Table S1 — Prediction performance of TMhhcp on the 4 protein chains for which MEMPACK failed to predict any residue contact. (DOC) [file pone.0026767.s002.doc]

**Table S1.** Prediction performance of MEMPACK and TMhhcp on the 17 protein chains.

| **Predictor** | **Accuracy (%)** | **Coverage (%)** | **Accuracy (δ=4) (%)** |
| --- | --- | --- | --- |
| TMhhcp1^a^ | 53.1 | 6.7 | 86.3 |
| TMhhcp2^b^ | 51.1 | 6.4 | 82.8 |
| MEMPACK1^a^ | 44.7 | 12.9 | 77.8 |
| MEMPACK2^b^ | 42.7 | 21.5 | 75.4 |

^a^Residue contact definition 1 (i.e. DEF1)

^b^Residue contact definition 2 (i.e. DEF2)
