# Supplementary material for: Predicting Residue-Residue Contacts and Helix-Helix Interactions in Transmembrane Proteins Using an Integrative Feature-Based Random Forest Approach
Source: PLoS One. 2011 Oct 28;6(10):e26767. doi: 10.1371/journal.pone.0026767 (PMC3203928; doi:10.1371/journal.pone.0026767)
Supplement: Table S2 — Prediction performance of TMhhcp on the 4 protein chains for which MEMPACK failed to predict any residue contact. (DOC) [file pone.0026767.s003.doc]

**Table S2.** Prediction performance of TMhhcp on the four protein chains for which MEMPACK failed to predict any residue contact.

|  | **Accuracy (%)** | | **Coverage (%)** | | **Accuracy (δ=4) (%)** | |
| --- | --- | --- | --- | --- | --- | --- |
| **Protein chain** | **TMhhcp1^a^** | **TMhhcp2^b^** | **TMhhcp1^a^** | **TMhhcp2^b^** | **TMhhcp1^a^** | **TMhhcp2^b^** |
| 3EAMA | 37.5 | 18.8 | 6.5 | 4.1 | 100 | 100 |
| 3K3FA | 30 | 56.7 | 3.2 | 5.8 | 90 | 96.7 |
| 3KLYA | 15.4 | 23.1 | 1.9 | 3.0 | 38.5 | 46.2 |
| 3M73A | 25 | 27.8 | 2.8 | 3.0 | 77.8 | 88.9 |

^a^Residue contact definition 1 (i.e. DEF1)

^b^Residue contact definition 2 (i.e. DEF2)
