# Supplementary material for: Predicting Residue-Residue Contacts and Helix-Helix Interactions in Transmembrane Proteins Using an Integrative Feature-Based Random Forest Approach
Source: PLoS One. 2011 Oct 28;6(10):e26767. doi: 10.1371/journal.pone.0026767 (PMC3203928; doi:10.1371/journal.pone.0026767)
Supplement: Table S3 — The selected features based on the residue contact definition DEF1. (DOC) [file pone.0026767.s004.doc]

**Table S3.** The selected features based on the residue contact definition DEF1.

Features 1-360 represent the evolutionary profiles of the residue pair and their surrounding residues. Feature 361 represents the transmembrane helix numbers. Feature 362 represents relative distance of two residues. Feature 363 represents the sequence distance of two residues respectively. Features 364-381 represent the conservation scores. Features 382-390 represent the MI scores. Features 391-399 represent the MCBASC scores. Features 340-348 represent the OMES scores.

| feature | score |
| --- | --- |
| 363 | 100 |
| 386 | 100 |
| 362 | 100 |
| 377 | 69 |
| 173 | 42 |
| 294 | 38 |
| 147 | 28 |
| 59 | 24 |
| 174 | 22 |
| 145 | 18 |
| 339 | 17 |
| 291 | 16 |
| 194 | 16 |
| 240 | 16 |
| 94 | 16 |
| 261 | 15 |
| 61 | 13 |
| 310 | 11 |
| 5 | 11 |
| 136 | 10 |
| 375 | 10 |
| 368 | 9 |
| 81 | 9 |
| 317 | 9 |
| 76 | 9 |
| 56 | 8 |
| 333 | 7 |
| 139 | 7 |
| 208 | 7 |
| 231 | 7 |
| 336 | 7 |
| 230 | 6 |
| 16 | 6 |
| 366 | 6 |
| 23 | 5 |
| 345 | 5 |
| 216 | 5 |
| 288 | 5 |
| 10 | 4 |
| 140 | 4 |
| 248 | 4 |
| 121 | 4 |
| 205 | 4 |
| 167 | 3 |
| 400 | 3 |
| 8 | 3 |
| 201 | 3 |
| 238 | 3 |
| 306 | 3 |
| 297 | 2 |
| 32 | 2 |
| 128 | 2 |
| 316 | 2 |
| 189 | 2 |
| 156 | 2 |
| 46 | 2 |
| 305 | 2 |
| 17 | 2 |
| 320 | 2 |
| 11 | 2 |
| 329 | 1 |
| 72 | 1 |
| 237 | 1 |
| 99 | 1 |
| 301 | 1 |
| 292 | 1 |
| 255 | 1 |
| 311 | 1 |
| 75 | 1 |
| 144 | 1 |
| 196 | 1 |
| 296 | 1 |
| 165 | 1 |
| 22 | 1 |
| 239 | 1 |
| 45 | 1 |
| 325 | 1 |
| 41 | 1 |
| 70 | 1 |
| 187 | 1 |
| 133 | 1 |
| 234 | 1 |
| 260 | 1 |
| 318 | 1 |
| 228 | 1 |
| 359 | 1 |
| 88 | 1 |
| 241 | 1 |
| 355 | 1 |
